# Remarkable immune and clinical value of novel ferroptosis-related genes in glioma

**DOI:** 10.1038/s41598-022-17308-7

**Published:** 2022-07-27

**Authors:** Xiaoyan Gao, Jiazheng Zhao, Litao Jia, Qiushi Zhang

**Affiliations:** 1 Department of Neurosurgery, The Fourth Hospital of Hebei Medical University, 12 Health Road, Shijiazhuang, 050011 Hebei People’s Republic of China; 2 Department of Orthopedics, The Fourth Hospital of Hebei Medical University, Shijiazhuang, Hebei People’s Republic of China; 3 Department of CT Scan, The Fourth Hospital of Hebei Medical University, Shijiazhuang, Hebei People’s Republic of China

**Keywords:** Cancer genetics, Neurology

## Abstract

Ferroptosis is a neoteric model of regulated cell death that shows great potential for the understanding of tumor immunology and as a target for therapy. The present study aimed to identify ferroptosis-related differentially expressed genes (DEGs) in glioma and to explore their value through systematic analysis. Ferroptosis-related DEGs were identified through the Gene Expression Omnibus database in combination with the FerrDb database and analyzed in the Genotype-Tissue Expression database and The Cancer Genome Atlas database. Possible signaling pathways involved were explored by construction of enrichment analysis and protein–protein interaction of these DEGs. Potential regulation of the immune microenvironment, immune checkpoint and chemokine was postulated by immune analysis. A prognosis model for glioma was developed using survival analysis, exhibited by the nomogram and evaluated by the calibration curve. The prognostic value of the model was validated by using an independent cohort. A total of 15 ferroptosis-related DEGs were identified, including 7 down-regulated and 8 up-regulated, with ATP6V1G2, GABARAPL1 and GOT1 as hub genes. The expression of all 3 hub genes was positively correlated with T follicular helper cells and natural killer CD56bright cells. These hub genes were negatively correlated with the macrophage cell type as well as B7H3, PDCD1, LAG3 and CXCL16, CXCR4, CCR5. Low expression of all 3 hub genes was associated with poor prognosis in glioma cases. ATP6V1G2 might be an independent prognostic factor and, as such, a high-precision prognostic model of glioma was constructed. We identified novel ferroptosis-related genes with clinical value in glioma and revealed their possible tumor immune relevance. Furthermore, in glioma, we pinpointed underlying critical elements of the chemokine, immune microenvironment and immune checkpoint, and were able to develop a predictive model of prognosis.

## Introduction

Ferroptosis is a neoteric model of regulated cell death (RCD) that relies on lipid peroxidation and iron catalysis to induce the accumulation of reactive oxygen species^1^. Growing evidence suggests that ferroptosis is integrally linked to the development, progression and suppression of cancer, as well as exhibiting great potential in tumor immunology and therapy^2,3^. The genes involved in ferroptosis are considered significant in relation to glioma^4^.

Gliomas are the most common primary tumors of the brain^5^ which are deemed to originate from progenitor cells or neuroglial stem and are classified as grades I-IV depending on the degree of malignancy^6^. As a complicated and heterogeneous tumor, the pathogenesis and regulation of glioma encompass multiple pathways involving the immune microenvironment, non-coding RNA, and metabolic reprogramming^7,8^. Furthermore, the prognosis of glioma is not favourable, with age being generally regarded as one of the risk factors^9^ and, in recent years, IDH mutation and 1p/19q deletion have also been included as prognostic indicators^10^. Emerging therapeutic strategies are urgently needed, and studies related to ferroptosis provide new insights into the treatment of glioma.

In this study we identified novel ferroptosis-related genes in glioma through the Gene Expression Omnibus (GEO) database in combination with the FerrDb database and analyzed them in the Genotype-Tissue Expression (GTEx) database and the Cancer Genome Atlas (TCGA) database. Possible signaling pathways involved were explored by construction of protein–protein interaction (PPI) and enrichment analysis. Potential regulation of the immune microenvironment, immune checkpoint and chemokine in glioma was postulated by immune analysis. A new independent prognostic factor was located by survival analysis and used to develop a highly accurate prognostic model. Ultimately, the prognostic value was validated in the Chinese Glioma Genome Atlas (CGGA) database.

## Materials and methods

### Data sources

**Figure 1:**
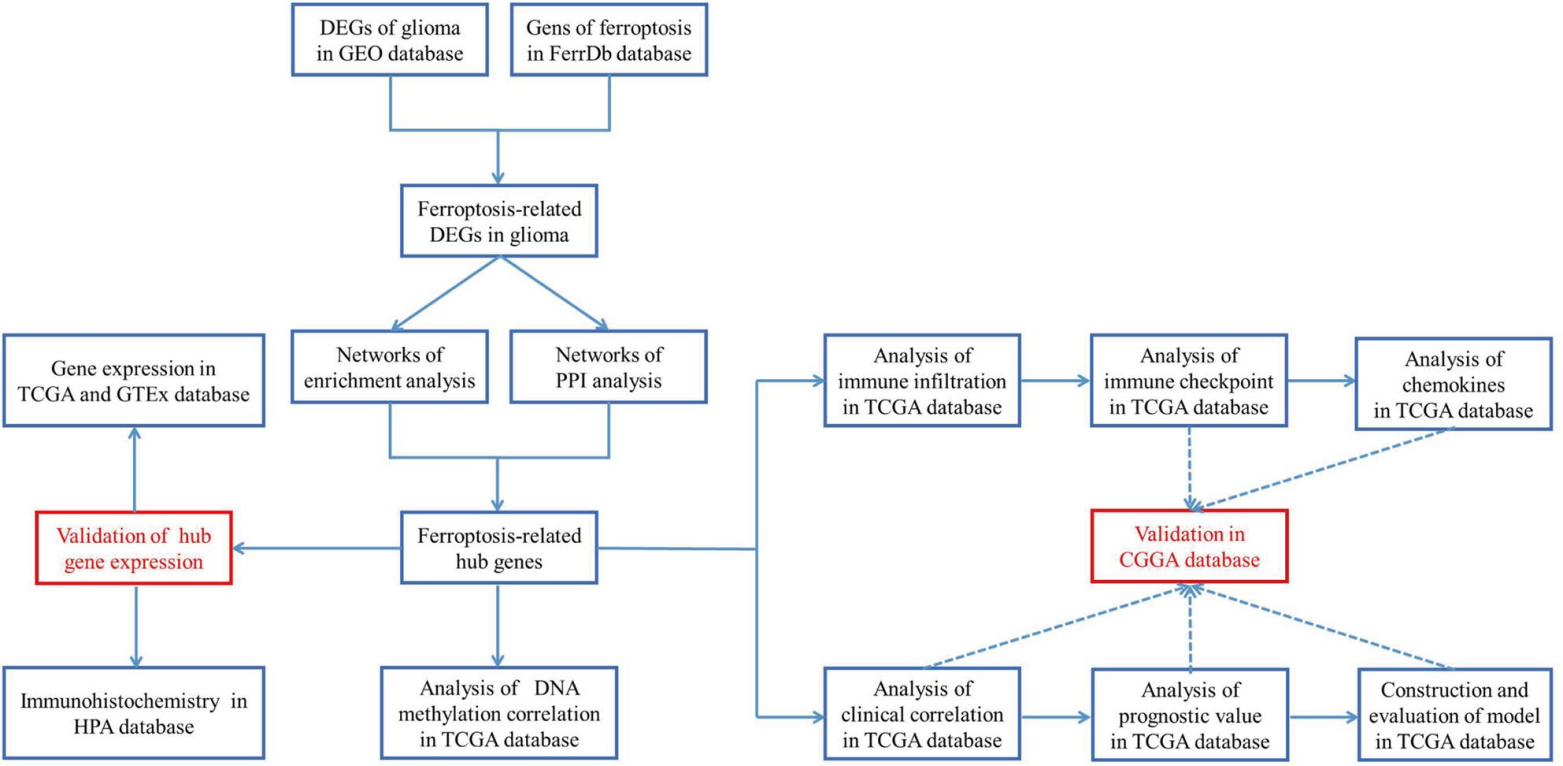
The flow chart of the present study design.

The flow chart of the present study design is demonstrated in Fig. 1. We downloaded the raw probe-level data (CEL files) for the GSE4290 and GSE50161 datasets from the GEO database (https://www.ncbi.nlm.nih.gov/geo/) with a total of 274 glioma samples and 36 non-tumor samples. The platform for both GSE4290 and GSE50161 is GPL570, with GSE4290 comprising 157 glioma samples and 23 non-tumor samples, and GSE50161 comprising 117 tumor samples and 13 non-tumor samples. From the TCGA database (https://www.cancer. gov/about-nci/organization/ccg/research/structural-genomics/tcga), we obtained a total of 694 samples with RNA-seq data and clinical data, including 689 glioma samples and 5 paraneoplastic samples. In addition, we collected RNA-seq data from a total of 1152 normal brain tissue samples from the GTEx database (https://www.gtexportal.org/). RNA-seq data in FPKM format were converted to TPM format and log2 transformed. FerrDb (http://www.zhounan.org/ferrdb) is the first manually managed ferroptosis database covering regulatory factors and molecular markers for ferroptosis and ferroptosis-related diseases^11,12^. Ferroptosis-related genes are defined as the gene ensemble consisting of Drivers, Suppressors of ferroptosis and Markers of ferroptosis process. We acquired all currently known ferroptosis-related genes from the FerrDb database, totaling 259. RNA-seq and survival information from the CGGA database (http://www.cgga.org.cn) of 313 glioma samples were used for validation. All methods were carried out in accordance with relevant guidelines and regulations. The above data were all obtained from public databases and did not involve informed consent from patients.

### Pretreatment and differential analysis

The raw probe-level data (CEL files) were read using the affy package of R^13^. The robust multichip averaging (RMA) method was applied for preprocessing, including correction of background, standardization of data and calculation of expression. Missing values were added by the k-Nearest Neighbor (KNN) method. Differentially expressed genes (DEGs) were identified using the limma package^14^ with the filter that met an adjusted *P* value (false discovery rate, FDR) < 0.05 and |log fold change (logFC)| > 1. Subsequently, ferroptosis-related DEGs were obtained by taking the intersection of DEGs and all ferroptosis-related genes. The ComplexHeatmap package^15^ and the ggplot2 package were used for visualization.

### Functional enrichment analysis and gene regulatory network construction

The clusterProfiler package was used for Gene Ontology (GO) enrichment analysis, Kyoto Encyclopedia of Genes and Genomes (KEGG) pathway analysis^16^ and Gene Set Enrichment Analysis (GSEA), and the org.Hs.eg.dborg.Hs.eg.db package was used for gene ID conversion. Results were considered statistically significant at FDR < 0.05. Interactions in DEGs were predicted using the Search Tool for the Retrieval of Interacting Genes/Proteins (STRING) database (https://string-db.org/)^17^ with a combined score > 0.15. Cytoscape is an open-source software for network analysis and visualization^18^, which we used to build the PPI network.

### Molecular correlation analysis

High and low expression groups were classified according to the upper and lower quartiles of hub gene expression, and RNA-seq data of 689 glioma samples from TCGA was used for immune analysis by ssGSEA algorithm in the GSVA package^19^. A total of 7 popular immune checkpoint genes (ICGs)^20,21^, 24 immune cell types which were major components of the tumor microenvironment (TME)^22^, 30 primary chemokines and related receptors were integrated into the study. In addition, in the TCGA database of glioma samples, DNA methylation correlation of hub gene was evaluated by analyzing the association of gene expression and the Beta values corresponding to methylation probes. The statistical method adopted was the Spearman correlation analysis and results were considered statistically significant at *P* < 0.05.

### Clinical correlation and survival analysis

RNA-seq data of 694 samples from TCGA and 1152 normal samples from the GETx were handled uniformly using the Toil process^23^, validated for hub genes expression using the wilcoxon rank sum test, analyzed for diagnostic efficacy using the pROC package, and visualized using the ggplot2 package. Dunn's multiple comparison test was performed after retaining samples with World Health Organization (WHO) tumor grade information and removing duplicates from the 689 TCGA glioma samples, and then visualized with the ggplot2 package. In addition, the median gene expression was used as the cut-off value to divide the low and high expression groups, and overall survival (OS) was chosen as the prognostic parameter. After keeping samples with survival information and excluding duplicates from the 689 glioma samples, survival analyses^24,25^ were carried out using survival package, including log-rank test, univariate Cox regression and multivariate Cox regression, and the survminer package was used for visualization. The results of all the above statistical analyses were considered statistically significant at *P* < 0.05.

### Prognostic model construction and evaluation

The gene classified as the independent prognostic factor was validated internally and externally using the timeROC package in samples from the TCGA and CGGA databases, respectively. Subsequently, the statistically significant results of the multivariate Cox regression analysis were incorporated into the construction of a nomogram^26^, and the model was built by summing the prognostic factors for glioma to predict the 1-year, 2-year and 3-year survival probability of patients. Corresponding calibration curves were developed to assess the accuracy of the model. Ultimately, relying on an independent glioma cohort from the CGGA database, the resulting model was validated by constructing the time-dependent receiver operating characteristic (ROC) curve and decision curve analysis (DCA) figure. The rms package was used for visualization.

### Statistical analysis

The statistical analysis was conducted using R software version 3.6.3, STRING website version 11.0 and Cytoscape software version 3.8.2.

## Results

### Ferroptosis-related DEGs identification

**Figure 2:**
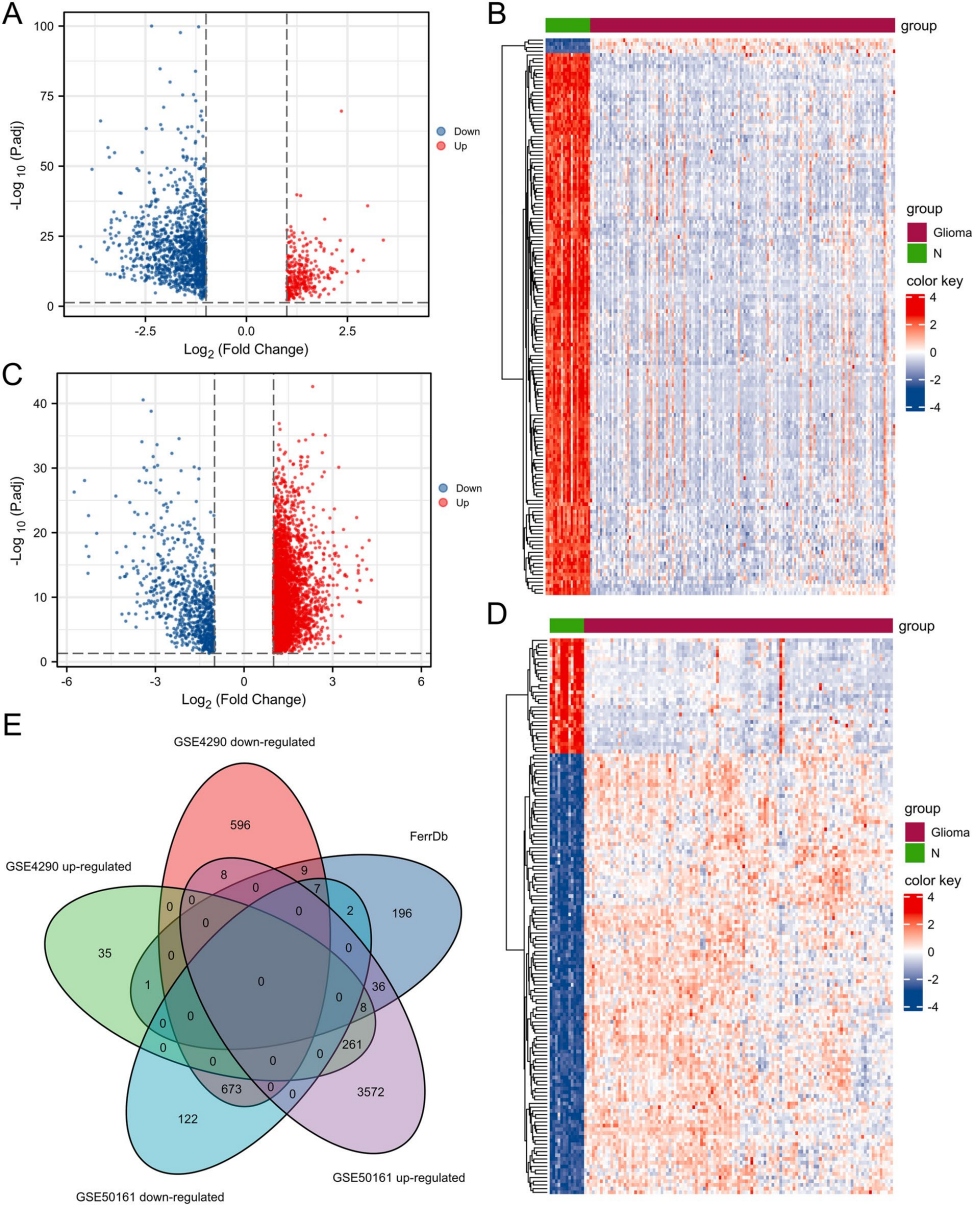
Identification of ferroptosis-related DEGs in GEO database. Volcano plots and heatmaps of DEGs in GSE4290 dataset (**A**, **B**, respectively) and GSE50161 dataset (**C**, **D**, respectively). The Venn diagram (**E**) of the intersection among up-regulated DEGs, down-regulated DEGs and ferroptosis-related genes.

A total of 1598 DEGs were identified in the GSE4290 dataset, of which 305 were up-regulated and 1293 were down-regulated. Furthermore, 4689 DEGs were identified in the GSE50161 dataset, of which 3885 were up-regulated and 804 were down-regulated. The volcano plot covered all DEGs in each dataset and the heatmap included the top 150 DEGs in each dataset ranked by FDR (Fig. 2A-D). The DEGs up-regulated in both datasets were intersected with 259 ferroptosis-related genes, 8 in total, namely IDH1, CD44, CAV*1*, DDIT4, TXNIP, VEGFA, RRM2 and TP53. The DEGs down-regulated in both datasets were intersected with 259 ferroptosis-related genes, 7 in total, namely GLS2, GOT1, GABARAPL1, FBXW7, ENPP2, ATP6V1G2 and RGS (Fig. 2E). Altogether, 15 ferroptosis-related DEGs of glioma were identified.

### GO and KEGG enrichment analyses of DEGs

**Figure 3:**
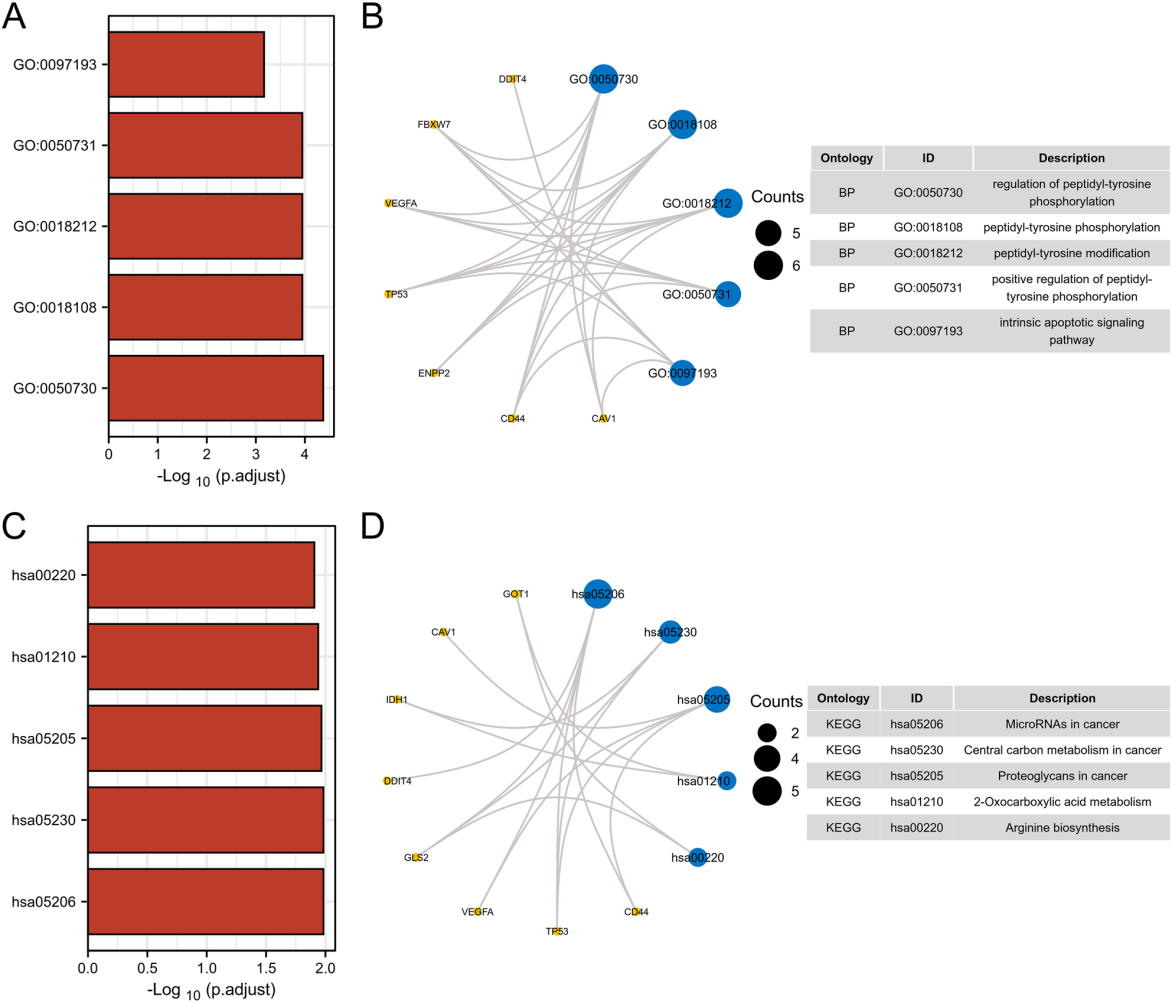
Enrichment analysis of ferroptosis-related genes. Using the top 5 significant enriched terms (**A**) to construct the GO visualization network (**B**). Using the top 5 significant enriched pathways (**C**) to construct the KEGG visualization network (**D**).

GO enrichment analysis was performed on the 15 DEGs, and the top 5 significantly enriched terms were filtered out and visualized in a network (Fig. 3A, B). The results revealed that these genes were mainly enriched in the biological process (BP) category and functioned in peptidyl-tyrosine phosphorylation, peptidyl-tyrosine modifications and intrinsic apoptotic signaling. These 15 ferroptosis-related DEGs were analyzed for KEGG enrichment and the top 5 significantly enriched pathways were screened for visual network construction (Fig. 3C, D). Corresponding genes were notably associated with microRNAs in cancer, central carbon metabolism in cancer, proteoglycans in cancer, 2-Oxocarboxylic acid metabolism and arginine biosynthesis.

### Hub genes identification and analyses

**Figure 4:**
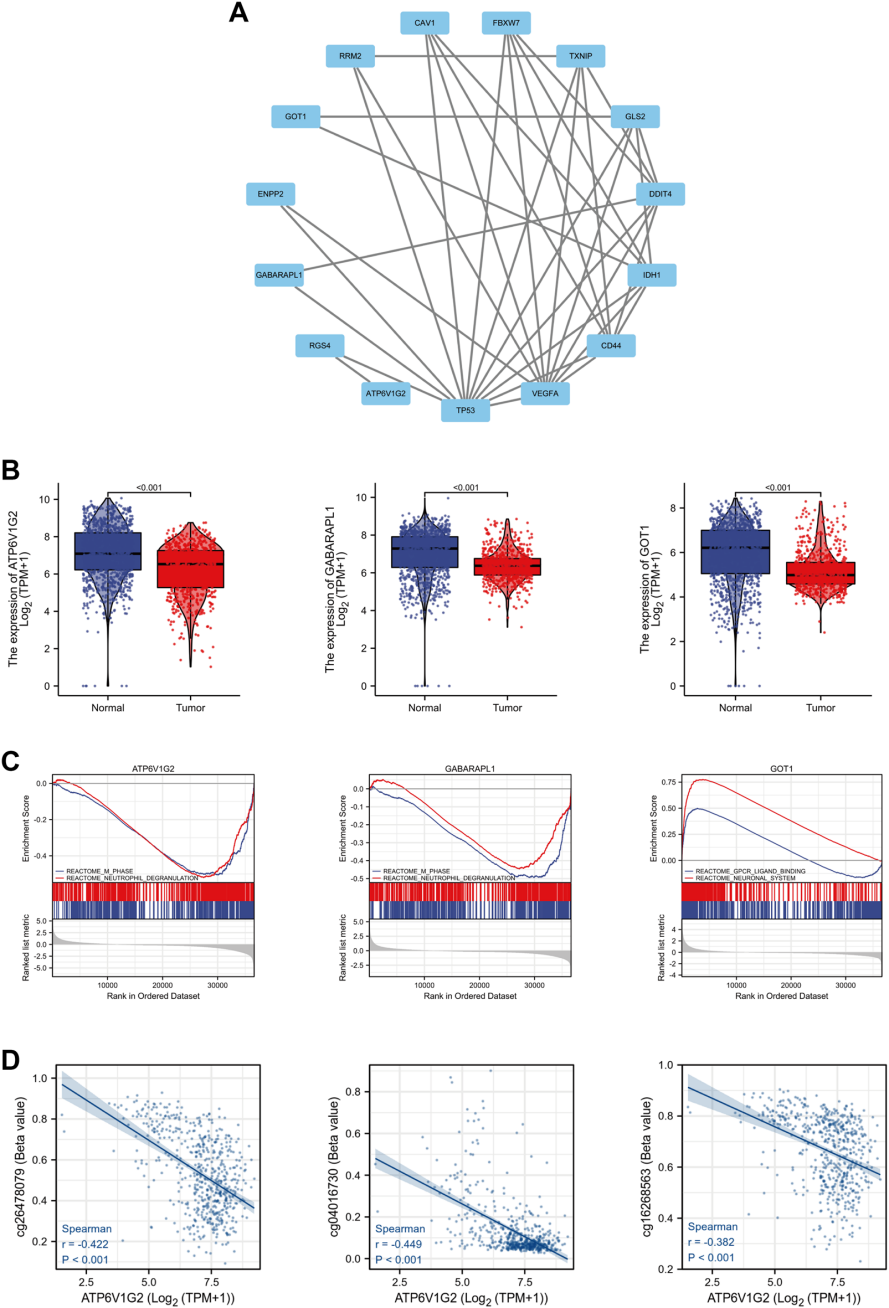
Hub genes identification and analyses. (**A**) The PPI network of ferroptosis-related genes. (**B**) Validation of hub genes expression in TCGA database and GTEx database. (**C**) GSEA of hub genes expression in TCGA database. (**D**) DNA methylation correlation analysis in TCGA database.

We predicted interactions among the ferroptosis-related DEGs using STRING and subsequently constructed a PPI network containing 15 nodes and 37 edges using Cytoscape (Fig. 4A). Three (ATP6V1G2, GABARAPL1 and GOT1) of the 15 genes, which were rarely reported in glioma, were identified as hub genes and subjected to further analysis. The expression of the hub genes was verified in 694 samples from TCGA and 1152 samples from GTEx, and all three genes were significantly under-expressed in glioma compared to normal ones (all *P* < 0.05) (Fig. 4B). Subsequently, 689 tumor samples were divided into low and high expression groups based on the median expression of each of the three hub genes, respectively, for GSEA (Fig. 4C), which manifested significant differences in the enrichment of MSigDB Collection (FDR < 0.05). Significant-enriched gene sets were ranked based on normalized enrichment score (NES) values. For both ATP6V1G2 and GABARAPL1, the top-two most significant-enriched gene sets were M phase and neutrophil degranulation. The top-two most significant-enriched gene sets for GOT1 were GPCR-ligand binding and neuronal system. Additionally, in terms of ATP6V1G2, the expression relationship between ATP6V1G2 and multiple DNA methylation probes demonstrated significant negative correlations (all *P* < 0.05, r < − 0.3) in the TCGA database of glioma samples (Fig. 4D).

### Association of hub genes expression and immune cells infiltration

**Figure 5:**
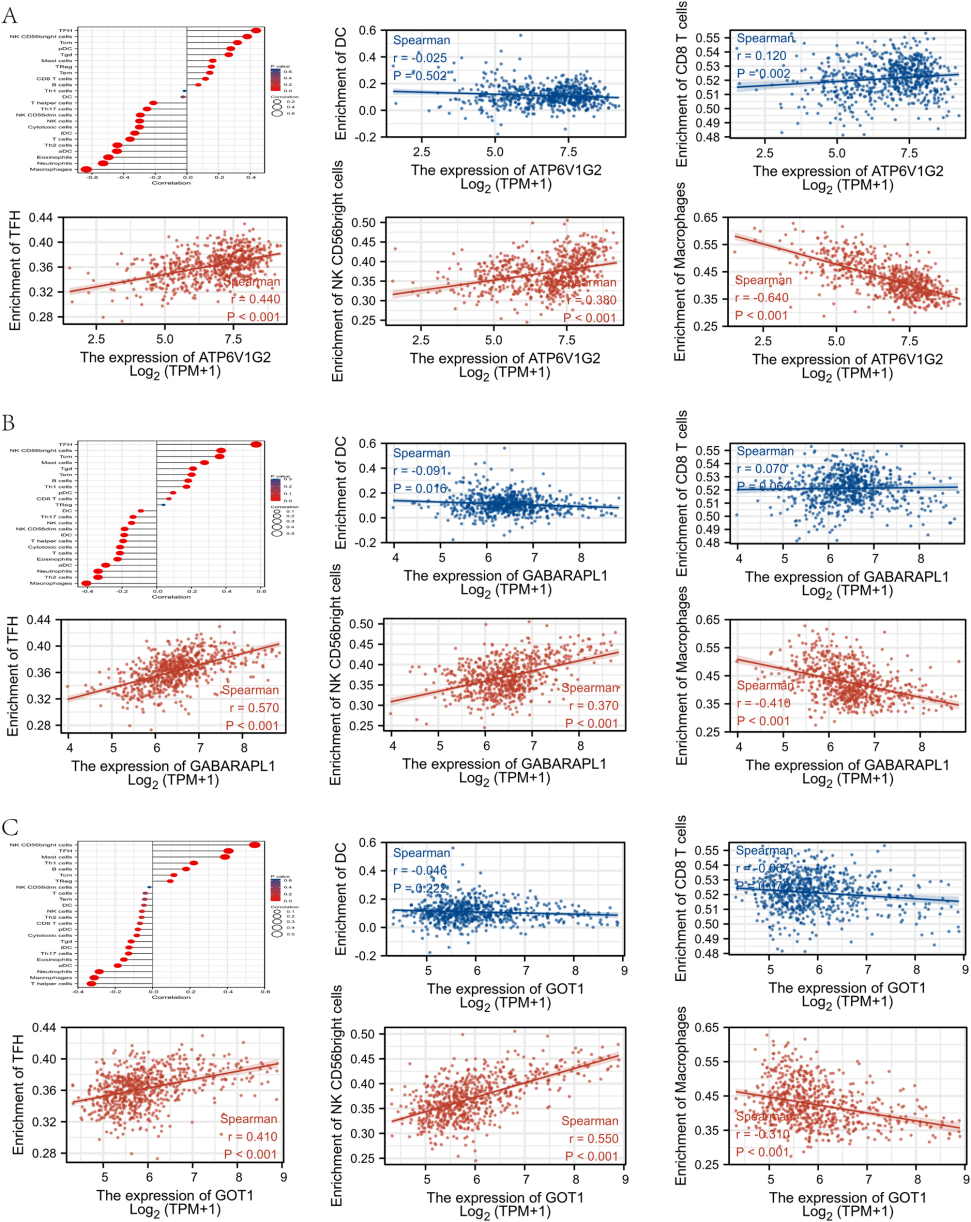
Visualization of the immune infiltration analysis in TCGA database. Association of ATP6V1G2 expression (**A**), GABARAPL1 expression (**B**), GOT1 expression (**C**) with immune cells infiltration, respectively.

Glioma samples (n = 689) excluding paraneoplastic ones from 694 samples derived from TCGA were used to explore the potential association between hub gene expression and immune cell infiltration. Similarly, the expression of all 3 hub genes were positively correlated (all *P* < 0.05, r > 0.3) with T follicular helper (Tfh) cells and natural killer (NK) CD56bright cells, while being negatively correlated (all *P* < 0.05, r < − 0.3) with macrophages. However, the correlation with dendritic cell (DC) and CD8^+^ T cell was either absent or slight (Fig. 5A-C).

### Association of hub genes expression and ICGs

**Figure 6:**
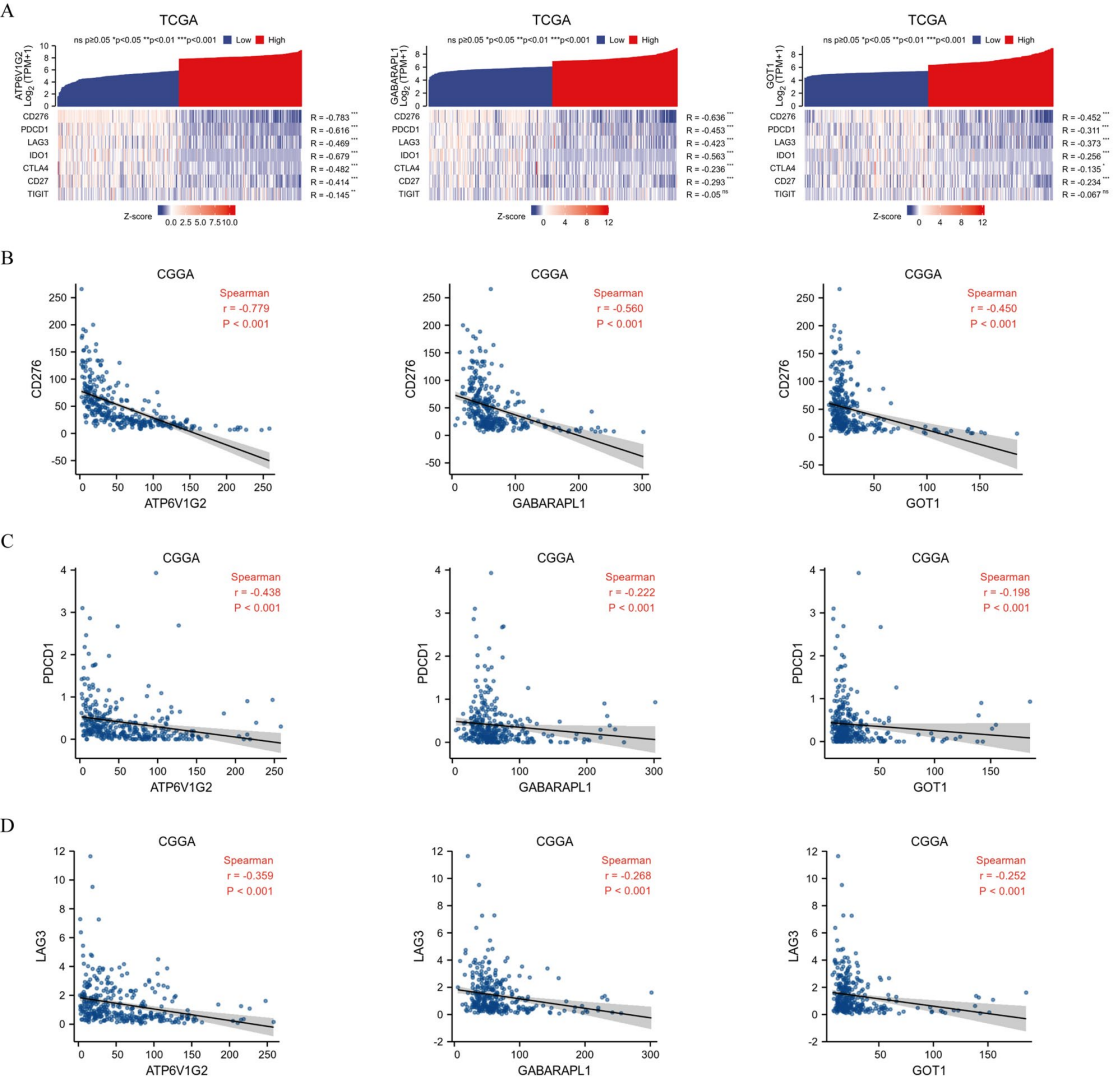
Visualization of the ICGs analysis. Association of hub genes with ICGs in TCGA database (**A**). Association of hub genes with CD276 (**B**), PDCD1 (**C**) and LAG3 (**D**) in CGGA database.

In the TCGA database, co-expression analysis of hub genes with ICGs showed a regular pattern, with CD276 (B7H3), PDCD1, LAG3 being negatively correlated (all *P* < 0.05, r < − 0.3) with all 3 hub genes in glioma, while TIGIT was not associated robustly (Fig. 6A). CD276, as the most strongly associated ICG, was selected among 313 glioma samples from the CGGA database to verify the relationship with the hub genes. The results showed that the expression of ATP6V1G2, GABARAPL1 and GOT1 was negatively correlated with CD276 expression (all *P* < 0.05, r < − 0.3) (Fig. 6B). Besides, correlation analysis of hub genes with PDCD1 (Fig. 6C) and LAG3 (Fig. 6D) in the CGGA database was consistent with the outcomes in the TCGA database.

### Association of hub genes expression and chemokines

**Figure 7:**
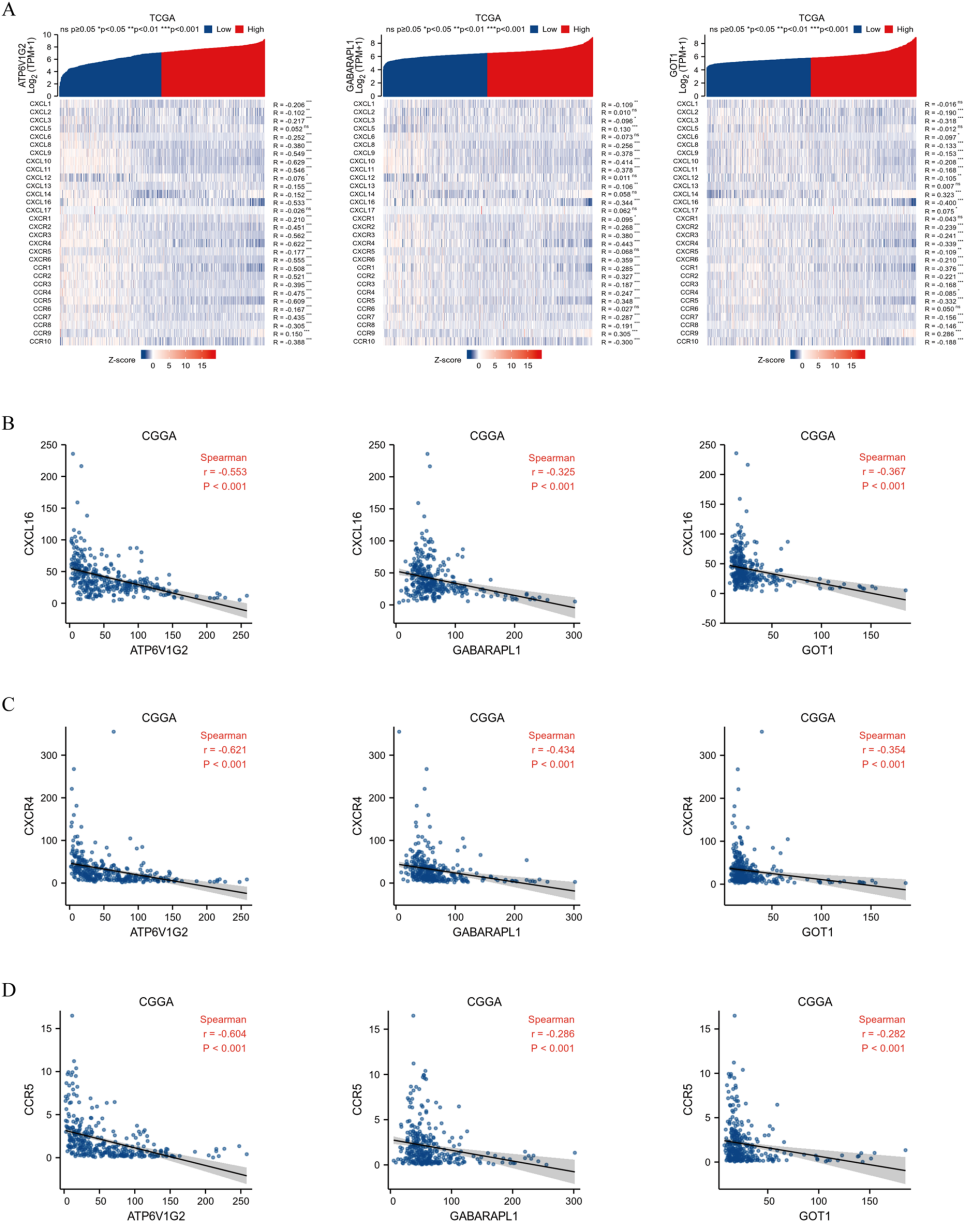
Visualization of the chemokines analysis. Association of hub genes with chemokines and related receptors in TCGA database (**A**). Association of hub genes with CXCL16 (**B**), CXCR4 (**C**) and CCR5 (**D**) in CGGA database.

Chemokine family plays an essential role in regulating infiltration degree of immune cell. In the TCGA database, co-expression analysis of hub genes with chemokines and related receptors also showed a regular pattern, with CXCL16 as a chemokine as well as CXCR4 and CCR5 as receptors negatively correlated (all *P* < 0.05, r < − 0.3) with all hub genes in glioma (Fig. 7A). The validation outcomes of CXCL16 (Fig. 7B), CXCR4 (Fig. 7C) and CCR5 (Fig. 7D) in the CGGA database exhibited consistent negative correlation trends, suggesting that CXCL16, CXCR4 and CCR5 might act as potentially vital chemokine elements in glioma.

### Association of hub gene expression and clinical variables

**Figure 8:**
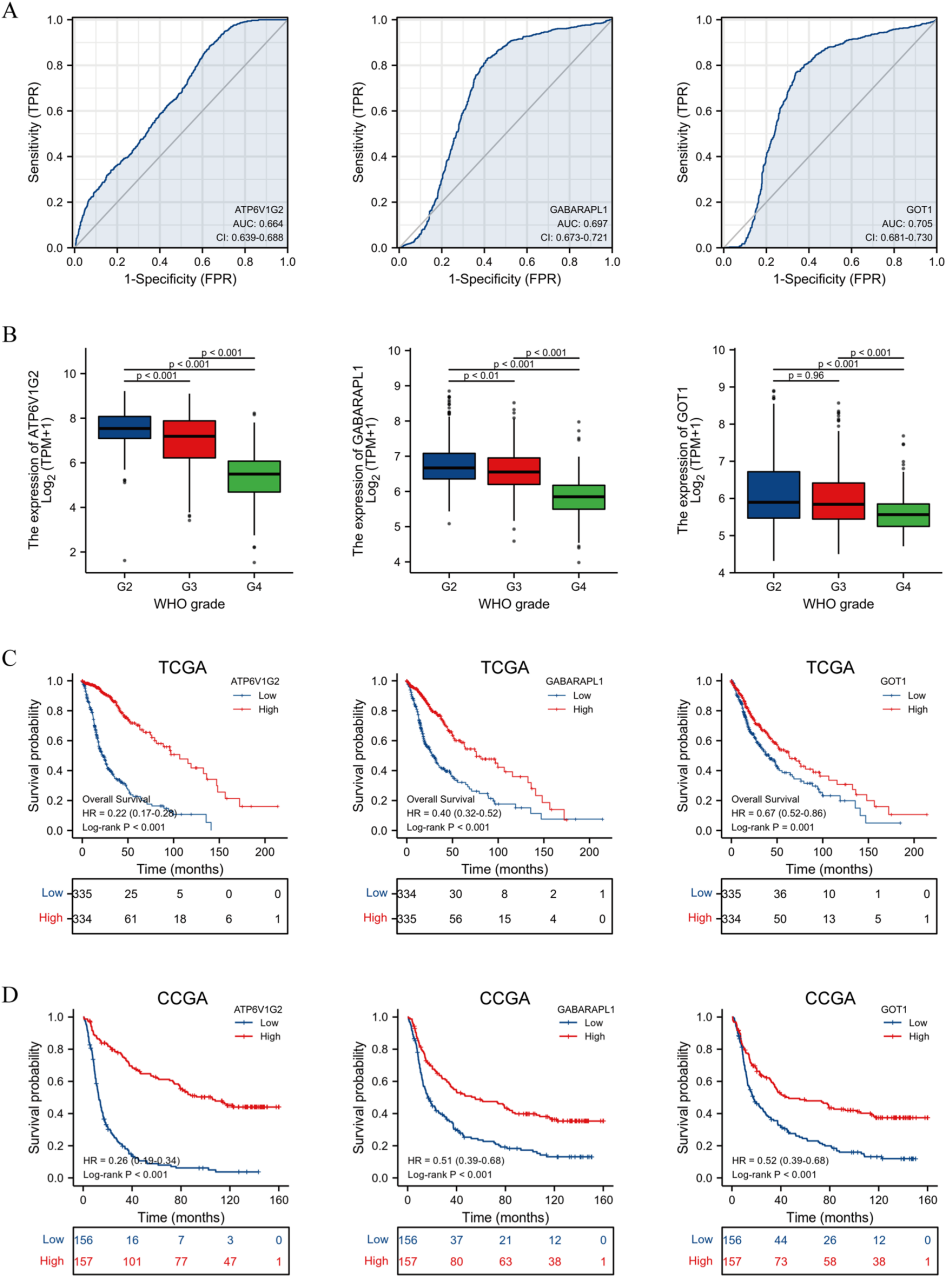
Association of hub genes expression and clinical variables. (**A**) ROC curves of hub genes in TCGA and GTEx database. (**B**) Expression of hub genes at different grades of glioma in TCGA database. (**C**) K-M curves of hub genes in TCGA database. (**D**) K-M curves of hub genes in CGGA database.

ROC curve analysis was performed on 1152 normal samples from GTEx, 689 glioma samples and 5 paraneoplastic samples from TCGA. The AUC values of ATP6V1G2, GABARAPL1 and GOT1 were 0.664, 0.697 and 0.705, respectively (all *P* < 0.05) (Fig. 8A). Subsequently, 612 glioma samples remained after excluding samples without tumor grade information and duplicates, and the potential association between hub gene expression and tumor grade was analyzed. Overall, the expression of ATP6V1G2, GABARAPL1 and GOT1 was significantly elevated in low-grade glioma compared to high-grade glioma (Fig. 8B). Likewise, 669 glioma samples remained after excluding samples with no survival information and duplicates, undergoing the log-rank test of hub genes expression and plotting the Kaplan–Meier (K–M) curves. The results suggested that high expression of ATP6V1G2, GABARAPL1 and GOT1 was associated with high survival probability as compared to low expression (Fig. 8C). Also, K–M curves in 313 gliomas sample through CGGA database showed that low expression of ATP6V1G2, GABARAPL1 and GOT1 was associated with poor prognosis (Fig. 8D), which was consistent with the results from the TCGA database.

### ATP6V1G2 was an independent prognostic factor for glioma patients

**Figure 9:**
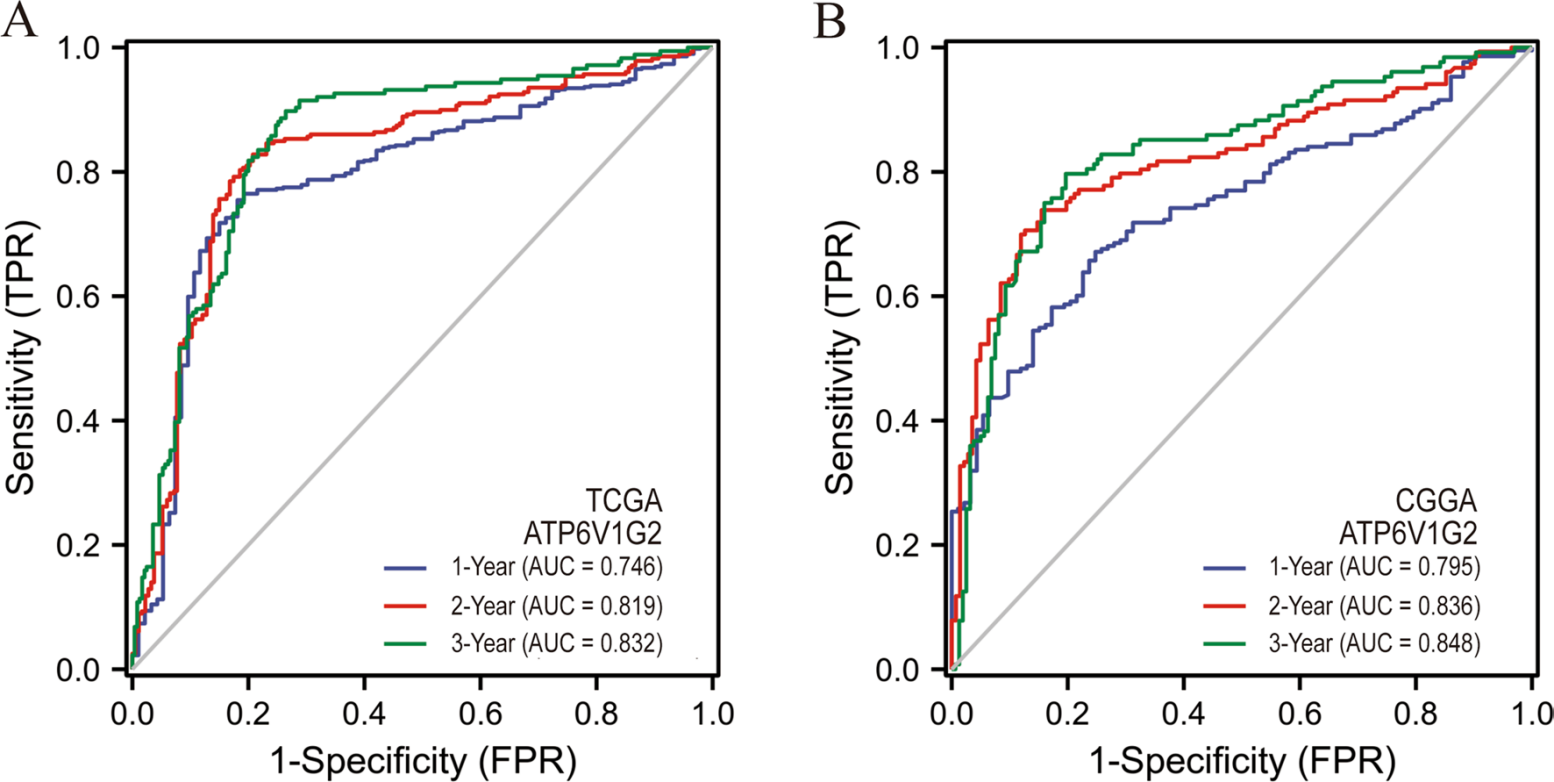
Validation of the ATP6V1G2 prognostic value. (**A**) The time-dependent ROC curve of ATP6V1G2 in TCGA database. (**B**) The time-dependent ROC curve of ATP6V1G2 in CGGA database.

The expression of ATP6V1G2, GABARAPL1, GOT1 along with common glioma characteristics (age, gender, race, tumor grade, 1p/19q status, IDH status) were included in the univariate Cox regression analysis. The results implied that advanced age, high-grade tumor, 1p/19q non-codeletion, IDH-non mutation, low expression of ATP6V1G2, GABARAPL1 and GOT1 might be poor prognostic factors for glioma patients. The above potential factors were further combined into the multivariate Cox regression analysis, and the results indicated that ATP6V1G2 might be an independent prognostic factor of glioma. Glioma patients with low ATP6V1G2 expression tended to suffer a shorter OS than those with high ATP6V1G2 expression (Table 1). Internal validation of the prognostic value for ATP6V1G2 was performed using 669 glioma samples from TCGA, with the time-dependent ROC curve showing high predictive efficacy of ATP6V1G2 (Fig. 9A). External validation was achieved using 313 glioma samples from CGGA, and the time-dependent ROC curve similarly validated the efficient predictive efficacy of ATP6V1G2 on glioma prognosis (Fig. 9B).

**Table 1:** Univariate and multivariate Cox regression analysis to identify prognostic factors for glioma patients.

Variables	Total (N)	Univariate analysis		Multivariate analysis	
		Hazard ratio (95% CI)	*P* value	Hazard ratio (95% CI)	*P* value
Age, years (> 60 vs. < = 60)	669	4.716 (3.609–6.161)	< 0.001	1.874 (1.382–2.540)	< 0.001
Gender (Male vs. female)	669	1.230 (0.955–1.585)	0.109		
Race (White vs. others)	657	1.240 (0.757–2.032)	0.393		
Tumor grade (G4&G3 vs. G2)	612	5.893 (4.015–8.648)	< 0.001	2.428 (1.588–3.714)	< 0.001
1p/19q codeletion (Non vs. codel)	663	4.635 (2.963–7.251)	< 0.001	1.168 (0.663–2.060)	0.591
IDH status (WT vs. Mut)	660	9.850 (7.428–13.061)	< 0.001	5.350 (3.508–8.158)	< 0.001
ATP6V1G2 (Low vs. high)	669	5.012 (3.730–6.735)	< 0.001	1.810 (1.164–2.816)	0.008
GABARAPL1 (Low vs. high)	669	2.502 (1.930–3.243)	< 0.001	0.710 (0.499–1.011)	0.058
GOT1 (Low vs. high)	669	1.497 (1.166–1.924)	0.002	1.055 (0.771–1.442)	0.739

### Construction and evaluation of the prognostic model for glioma patients

**Figure 10:**
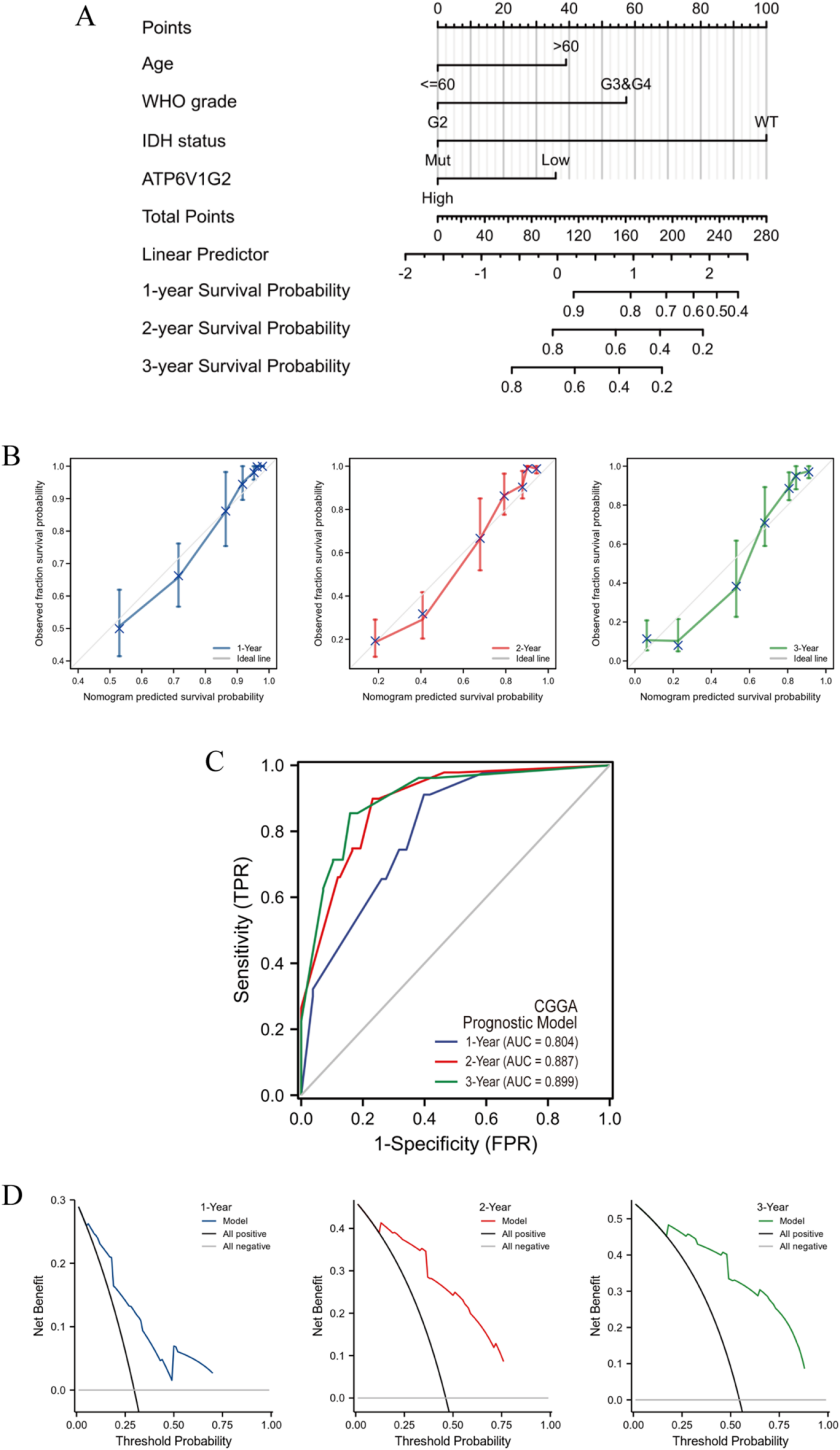
Visualization of the glioma prognostic model. Using the nomogram (**A**) and the calibration curve (**B**) to construct and evaluate the model in TCGA database, respectively. Using the time-dependent ROC curve (**C**) and the DCA figure (**D**) to validate the model in CGGA database.

The statistically significant results (age, tumor grade, IDH status, ATP6V1G2 expression) of the multivariate Cox regression analysis were incorporated into the construction of the nomogram (Fig. 10A). The values of the Points axis corresponding to each variable for glioma patients were summed and positioned in the Total Points axis, thus anticipating their 1-year, 2-year and 3-year survival probability. All the covariates in the nomogram satisfied proportional hazards assumptions and the global Schoenfeld test. The C-index of the model was 0.840 (95% CI 0.829–0.852). Additionally, in the calibration curve (Fig. 10B) the predicted 1-year, 2-year and 3-year survival probability matched closely with the ideal line, demonstrating the trend consistency and predictive accuracy of the prognostic model. Furthermore, validation using an independent cohort with 313 glioma samples from CGGA database showed that the prognostic model was highly effective in predicting the 1- year, 2- year and 3-year survival probability of patients (Fig. 10C). The predictive efficacy of this prognostic model was stronger than that of ATP6V1G2 alone. DCA plots likewise confirmed the nomogram combined with various features had considerable clinical application value (Fig. 10D).

## Discussion

Despite the continued discovery of genes relevant to glioma prognosis^27^ and the development of emerging therapies, the improvement in patient prognosis has been limited and the treatment outcome for some glioma patients has been suboptimal^28^. For three decades, most of the clinical strategies have functioned by targeting tumor cells to induce apoptosis^29^. However, cancer cells may undergo apoptotic escape, allowing them to generate resistance, leading to diminished therapeutic sensitivity and poor patient prognosis^30^. Distinct from apoptosis, ferroptosis as a neoteric form of RCD features unique biological and morphological characteristics and is gaining widespread attention in the treatment of refractory tumors^31^. Currently, studies of ferroptosis in glioma are sparse, and genetic determinants that play a crucial role need to be discovered urgently.

A total of 310 samples from the GEO database (274 glioma samples and 36 non-tumor samples) were analyzed for gene expression differences and intersected with ferroptosis-related genes from the FerrDb database, with 15 ferroptosis-related DEGs (8 up-regulated and 7 down-regulated) identified in glioma samples. We explored the possible biological effects of these DEGs through PPI network construction and enrichment analysis. The PPI network constructed covered all 15 DEGs and GO enrichment analysis indicated that they were intimately associated with peptidyl-tyrosine phosphorylation and peptidyl-tyrosine modifications. KEGG analysis revealed that ferroptosis-related DEGs were significantly enriched in 'microRNAs in cancer', 'central carbon metabolism in cancer' and 'proteoglycans in cancer'. MicroRNAs work in virtually all aspects of cancer biology, ranging from proliferation, invasion, metastasis and more^32^. Some microRNAs are considered to be particularly linked to the initiation, progression and prognosis of specific tumors^33^. As for glioma, multiple microRNAs have been recognized as regulators and prognostic markers, with miR-145, miR-31, miR-451, miR-143, miR-146a, and miR-126 being utilized as therapeutic molecules in primary and metastatic glioma^34^. In addition, malignant transformation of cells necessitates corresponding shifts in cellular metabolism to support growth and maintain survival. Transformations in carbon metabolism involving raised glutaminolysis, aerobic glycolysis, dysfunctional tricarboxylic acid cycle and pentose phosphate pathway occur within the tumor mass, thereby accelerating tumor progression and maintaining viability^35^. Proteoglycans serve as essential effector molecules on the cell surface and in the pericellular microenvironment, characterised by their polyhedric nature and capacity to interact with ligands and receptors which modulate neovascularization and tumor growth, resulting in a variety of roles in angiogenesis and cancer^36^. The discrete expression of proteoglycans and their interacting partners have been distinguished as specific for disease progression in varying types of tumor^37^. Besides, the significant negative correlations between the expression of ATP6V1G2 and multiple DNA methylation probes in glioma suggested that ATP6V1G2 might play an active role in the DNA methylation process. Although we have obtained various potential biological functions through 15 ferroptosis-related DEGs, due to the paucity of studies on ferroptosis, it is not feasible to confirm whether there is a clear relationship between these functions and ferroptosis to drive gene action in glioma, and such uncertain relationships can only be hypothesized and need to be further verified.

Ferroptosis and immunity interplay, with ferroptosis impacting the potency of tumor immunotherapy^38^, while ferroptosis is in turn mediated by CD8^+^ T cells^39^. Interestingly, in our study, immunoassay results for the hub genes were highly concordant, manifesting prominent positive correlations with NK CD56bright cell and Tfh cell types, prominent negative correlations with macrophage as well as B7H3, PDCD1, LAG3 and CXCL16, CXCR4, CCR5, which revealed for the first time the possible immunological effects of ATP6V1G2, GABARAPL1, and GOT1 in cancer. Given that, we hypothesized that NK CD56bright, Tfh, and macrophage cell types might be closely related to immune regulation in TME of glioma, and B7H3, PDCD1, LAG3 and CXCL16, CXCR4, CCR5 might be key targets for glioma immunotherapy. Lu et al. confirmed that activated NK cells, Tfh cells and macrophages could act as independent predictors of malignant transformation in low-grade glioma^40^, which validated our hypothesis. It is now generally accepted that immunology and immunotherapy are incredibly promising for therapeutic application in glioma, and ferroptosis is credited with playing a significant role in this^41^. Yee et al. proposed and proved that neutrophils could mediate ferroptosis and thus promote tumor necrosis to slow down the progression of glioblastoma^42^. Several studies have revealed the intimate association of ferroptosis-related genes with immune cells represented by neutrophils in glioma, but the specific mechanisms involved remain to be further clarified^43–45^. As for ICGs, B7H3 has been recognized as a biomarker of outstanding diagnostic and therapeutic profile in human glioblastoma^46^. The corresponding value of PDCD1 and LAG3 has also been demonstrated in successive glioma studies^47^. In addition, CXCL16 as a chemokine as well as CXCR4 and CCR5 as chemokine receptors were identified in our study as possible vital immunomodulatory factors in glioma. Chemokine family plays an essential role in regulating infiltration degree of immune cell. In summary, we aimed to investigate valuable immune elements that had commonality with hub gene expression in glioma from various perspectives, including immune infiltrating cells, ICGs and chemokines.

Subsequently, the results of the clinical correlation analysis suggested that the 3 hub genes, which were down-regulated in glioma samples, possessed certain diagnostic efficacy and were lowly expressed in high-grade tumors, as well as low expression accompanied by short OS. Combined with their high expression in non-glioma samples, we speculated that ATP6V1G2, GABARAPL1, and GOT1 might be suppressor genes for glioma. Moreover, we gradually performed log-rank test, univariate Cox regression analysis, and multivariate Cox regression analysis on the survival information, and constructed a prognostic model presented in the nomogram and conducted the accuracy test. The C-index (0.840) and the high fit of the calibration curve substantiated the superior latent value of the model. And eventually the nomogram was externally validated based on the ROC curves and DCA plots generated by an independent glioma cohort. In the construction of the model, we demonstrated that ATP6V1G2 could act as an independent prognostic factor for glioma patients and incorporated it into the prognostic model. ATP6V1G2 is recognized as an up-regulated biomarker of ferroptosis^48^, and as far as available studies indicated that ATP6V1G2 operated mainly in cardiovascular system diseases such as myocardial infarction^49^, adriamycin-induced cardiotoxicity^50^ and dilated cardiomyopathy^51^. This is the first study to confirm that ATP6V1G2 is tumor-related and may affect the prognosis of glioma patients. As for GABARAPL1 and GOT1, both are recognized as potential positive regulators of ferroptosis^52,53^. Su et al. established that GABARAPL1 inhibited prostate cancer metastasis by suppressing the PI3K/Akt pathway^54^. Zhang et al. enhanced the sensitivity of melanoma cells to ferroptosis inducers by increasing GOT1 expression^55^. Our study revealed the potential clinical relevance of these 3 ferroptosis-related hub genes, which might contribute to the prognosis prediction and precise treatment of patients with glioma.

Our study remains some limitations that need to undergo further validation in experiments and clinical cohorts. In addition to the present study, we also noticed that Zheng et al. proved the prognostic significance of the ferroptosis-related genes in low-grade glioma^56^. A growing number of ferroptosis-related genes with clinical value in glioma are also successively being identified^4,57^. Based on multiple databases, analyses and validation are thoroughly carried out in our study, and we believe that our findings are robust and might provide new insights.

## Conclusions

In conclusion, we identified novel ferroptosis-related genes with clinical value for glioma and revealed their possible tumor immune relevance. Furthermore, in glioma, we pinpointed underlying critical elements of the chemokine, immune microenvironment and immune checkpoint, and eventually achieved an efficient predictive model of prognosis.

## Data Availability

All data used, including gene expression, sequences and patient clinical information, are available from the GEO database (https://www.ncbi.nlm.nih.gov/geo/) (GSE4290, GSE50161), the TCGA database (https://www.cancer.gov/about-nci/organization/ccg/research/structural-genomics/tcga) and the CGGA database (http://www.cgga.org.cn). All methods were carried out in accordance with relevant guidelines and regulations. All data were obtained from public databases and was free of ethical issue or informed consent.
